# *N* ^2^,*N* ^2^,*N* ^4^,*N* ^4^-Tetra­ethyl-6-{2-[(*E*)-1-(4-nitro­phen­yl)ethyl­idene]hydrazino}-1,3,5-triazine-2,4-diamine

**DOI:** 10.1107/S1600536809025677

**Published:** 2009-07-11

**Authors:** Xiao-Ru Pan, Fang-Fang Jian

**Affiliations:** a Department of Chemistry and Chemical Engineering, Weifang University, Weifang 261061, People’s Republic of China; b Microscale Science Institute, Weifang University, Weifang 261061, People’s Republic of China

## Abstract

The title compound, C_19_H_28_N_8_O_2_, was prepared by the reaction of *N* ^2^,*N* ^2^,*N* ^4^,*N* ^4^-tetra­ethyl-6-hydrazino-1,3,5-triazine-2,4-diamine and 1-(4-nitro­phen­yl)ethanone in ethanol at room temperature. The mol­ecular conformation is stabilized by intra­molecular C—H⋯N hydrogen-bonding inter­actions. There are also inter­molecular N—H⋯O hydrogen bonds, and C—H⋯π and π–π inter­actions, which help to stabilize the crystal structure. The centroid–centroid distance is 3.6172 (10) Å between adjacent benzene and 1,3,5-triazine rings.

## Related literature

For the antimicrobial and anticancer applications of Schiff bases, see: Tarafder *et al.* (2000 ▶); Deschamps *et al.* (2003 ▶). For the ability of Schiff bases to form intramolecular hydrogen bonds by electron coupling between acid–base centers, see: Rozwadowski *et al.* (1999 ▶). For a related structure, see: Jian *et al.* (2006 ▶).
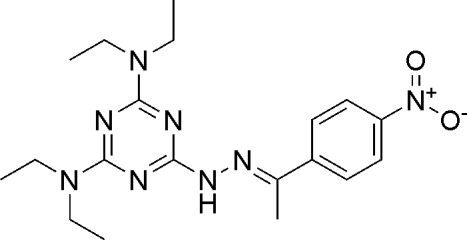


## Experimental

### 

#### Crystal data

C_19_H_28_N_8_O_2_
*M* *_r_* = 400.49
Monoclinic, 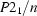

*a* = 12.333 (3) Å
*b* = 9.5286 (19) Å
*c* = 17.407 (4) Å
β = 92.12 (3)°
*V* = 2044.3 (7) Å^3^
*Z* = 4
Mo *K*α radiation
μ = 0.09 mm^−1^
*T* = 293 K
0.22 × 0.18 × 0.10 mm

#### Data collection

Bruker SMART CCD area-detector diffractometer
Absorption correction: none
19318 measured reflections
4667 independent reflections
4054 reflections with *I* > 2σ(*I*)
*R*_int_ = 0.018

#### Refinement

*R*[*F* ^2^ > 2σ(*F* ^2^)] = 0.036
*wR*(*F* ^2^) = 0.104
*S* = 1.05
4667 reflections
278 parameters
H atoms treated by a mixture of independent and constrained refinement
Δρ_max_ = 0.35 e Å^−3^
Δρ_min_ = −0.24 e Å^−3^

### 

Data collection: *SMART* (Bruker, 1997 ▶); cell refinement: *SAINT* (Bruker, 1997 ▶); data reduction: *SAINT*; program(s) used to solve structure: *SHELXS97* (Sheldrick, 2008 ▶); program(s) used to refine structure: *SHELXL97* (Sheldrick, 2008 ▶); molecular graphics: *SHELXTL* (Sheldrick, 2008 ▶); software used to prepare material for publication: *SHELXTL*.

## Supplementary Material

Crystal structure: contains datablocks global, I. DOI: 10.1107/S1600536809025677/at2835sup1.cif

Structure factors: contains datablocks I. DOI: 10.1107/S1600536809025677/at2835Isup2.hkl

Additional supplementary materials:  crystallographic information; 3D view; checkCIF report

## Figures and Tables

**Table 1:** Hydrogen-bond geometry (Å, °)

*D*—H⋯*A*	*D*—H	H⋯*A*	*D*⋯*A*	*D*—H⋯*A*
N3—H3*A*⋯O1^i^	0.868 (15)	2.491 (15)	3.2751 (15)	150.7 (13)
N3—H3*A*⋯O2^i^	0.868 (15)	2.474 (15)	3.2486 (15)	149.0 (13)
C2—H2*C*⋯N4	0.97	2.38	2.7252 (15)	100
C7—H7*A*⋯N5	0.97	2.39	2.7322 (16)	100
C15—H15*A*⋯N2	0.93	2.39	2.7128 (15)	100
C1—H1*A*⋯*Cg*2^ii^	0.96	2.91	3.7486 (16)	147
C7—H7*B*⋯*Cg*1^iii^	0.97	2.71	3.3835 (15)	127

Symmetry codes: (i) 
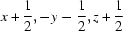
; (ii) 
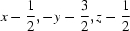
; (iii) 
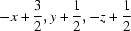
. *Cg*1 and *Cg*2 are centroids of the N4–N6/ C9–C11 and C14–C19 rings,  respectively.

**Fig. 1.**
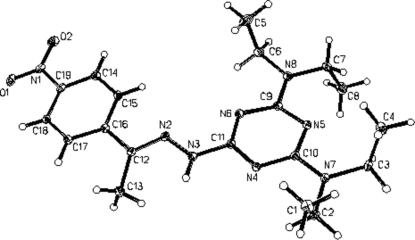
The structure of the title compound showing 30% probability displacement ellipsoids and the atom-numbering scheme.

## supplementary crystallographic information

### Comment

Schiff bases have antimicrobial (Tarafder *et al.*, 2000) and anticancer applications (Deschamps *et al.*, 2003). The recent growing interest in Schiff bases is also due to their ability to form intramolecular hydrogen bonds by electron coupling between acid-base centers (Rozwadowski *et al.*, 1999). The part of our research is to find Schiff base with higher biological activity, we sythesized the title compound (I) and report its crystal structure here.

In the crystal structure of compound (I) (Fig. 1), the dihedral angle formed by the C14 –C19 and N4–N6/C9–C11 rings was 10.76 (1)°. The C═N bond length [1.2910 (17) Å] is in agreement with that observed before (Jian *et al.*, 2006). There are intermolecular N—H···O hydrogen-bonds, C—H···π and π—π interactions to stabilize the crystal structure. The centroid–centroid distance iss 3.6172 (10) Å between the adjacent benzene and 1,3,5-triazine rings.

### Experimental

A mixture of N2,N2,N4,N4-tetraethyl-6-hydrazinyl-1,3,5-triazine-2,4-diamine (0.02 mol) and 1-(4-nitrophenyl)ethanone (0.02 mol) was stirred with ethanol (50 mL) at 298 K for 2 h, affording the title compound (6.40 g, yield 80.0%). Single crystals suitable for X-ray measurements were obtained by recrystallization from ethanol at room temperature.

### Refinement

The title compound, C_19_H_28_N_8_O_2_, was prepared by the reaction of *N*^2^,*N*^2^,*N*^4^,*N*^4^-tetraethyl-6-hydrazino-1,3,5-triazine-2,4-diamine and 1-(4-nitrophenyl)ethanone with ethanol at room temperature. The molecular conformation is stabilized by intramolecular C—H···N hydrogen-bonding interactions. There are also intermolecular N—H···O hydrogen bonds, and C—H···π and π–π interactions, which help to stabilize the crystal structure. The centroid–centroid distance is 3.6172 (10) Å between adjacent benzene and 1,3,5-triazine rings.

### Crystal data

**Table:**

C_19_H_28_N_8_O_2_	*F*(000) = 856
*M**_r_* = 400.49	*D*_x_ = 1.301 Mg m^−^^3^
Monoclinic, *P*2_1_/*n*	Mo *K*α radiation, λ = 0.71073 Å
Hall symbol: -P 2yn	Cell parameters from 4667 reflections
*a* = 12.333 (3) Å	θ = 3.2–27.5°
*b* = 9.5286 (19) Å	µ = 0.09 mm^−^^1^
*c* = 17.407 (4) Å	*T* = 293 K
β = 92.12 (3)°	Block, yellow
*V* = 2044.3 (7) Å^3^	0.22 × 0.18 × 0.10 mm
*Z* = 4	

### Data collection

**Table:**

Bruker SMART CCD area-detector diffractometer	4054 reflections with *I* > 2σ(*I*)
Radiation source: fine-focus sealed tube	*R*_int_ = 0.018
graphite	θ_max_ = 27.5°, θ_min_ = 3.2°
φ and ω scans	*h* = −16→15
19318 measured reflections	*k* = −12→12
4667 independent reflections	*l* = −22→22

### Refinement

**Table:**

Refinement on *F*^2^	Primary atom site location: structure-invariant direct methods
Least-squares matrix: full	Secondary atom site location: difference Fourier map
*R*[*F*^2^ > 2σ(*F*^2^)] = 0.036	Hydrogen site location: inferred from neighbouring sites
*wR*(*F*^2^) = 0.104	H atoms treated by a mixture of independent and constrained refinement
*S* = 1.05	*w* = 1/[σ^2^(*F*_o_^2^) + (0.0544*P*)^2^ + 0.6629*P*] where *P* = (*F*_o_^2^ + 2*F*_c_^2^)/3
4667 reflections	(Δ/σ)_max_ = 0.001
278 parameters	Δρ_max_ = 0.35 e Å^−^^3^
0 restraints	Δρ_min_ = −0.24 e Å^−^^3^

### Special details

**Table:**

Geometry. All e.s.d.'s (except the e.s.d. in the dihedral angle between two l.s. planes) are estimated using the full covariance matrix. The cell e.s.d.'s are taken into account individually in the estimation of e.s.d.'s in distances, angles and torsion angles; correlations between e.s.d.'s in cell parameters are only used when they are defined by crystal symmetry. An approximate (isotropic) treatment of cell e.s.d.'s is used for estimating e.s.d.'s involving l.s. planes.
Refinement. Refinement of *F*^2^ against ALL reflections. The weighted *R*-factor *wR* and goodness of fit *S* are based on *F*^2^, conventional *R*-factors *R* are based on *F*, with *F* set to zero for negative *F*^2^. The threshold expression of *F*^2^ > σ(*F*^2^) is used only for calculating *R*-factors(gt) *etc*. and is not relevant to the choice of reflections for refinement. *R*-factors based on *F*^2^ are statistically about twice as large as those based on *F*, and *R*- factors based on ALL data will be even larger.

### Fractional atomic coordinates and isotropic or equivalent isotropic displacement parameters (Å^2^)

**Table:**

	*x*	*y*	*z*	*U*_iso_*/*U*_eq_	
N6	0.86798 (7)	0.13524 (9)	0.14238 (5)	0.01620 (18)	
O2	0.61084 (7)	−0.21575 (10)	−0.26492 (5)	0.0282 (2)	
O1	0.71950 (7)	−0.38030 (9)	−0.29988 (5)	0.02543 (19)	
N3	0.97484 (7)	−0.06303 (10)	0.12715 (5)	0.01717 (19)	
N5	0.91414 (7)	0.28722 (9)	0.24899 (5)	0.01601 (19)	
N4	1.01161 (7)	0.07162 (9)	0.23260 (5)	0.01529 (18)	
N7	1.05089 (7)	0.22043 (9)	0.33480 (5)	0.01625 (19)	
N2	0.91687 (7)	−0.09648 (9)	0.06200 (5)	0.01611 (19)	
C19	0.75621 (8)	−0.27251 (11)	−0.18232 (6)	0.0168 (2)	
N1	0.69125 (7)	−0.29130 (10)	−0.25356 (5)	0.0200 (2)	
N8	0.77480 (7)	0.34066 (10)	0.16191 (5)	0.01862 (19)	
C15	0.79067 (8)	−0.14525 (11)	−0.06617 (6)	0.0180 (2)	
H15A	0.7718	−0.0751	−0.0320	0.022*	
C11	0.94887 (8)	0.05417 (11)	0.16864 (6)	0.0146 (2)	
C16	0.88343 (8)	−0.22754 (11)	−0.04984 (6)	0.0148 (2)	
C17	0.90831 (8)	−0.33498 (11)	−0.10124 (6)	0.0168 (2)	
H17A	0.9682	−0.3920	−0.0905	0.020*	
C10	0.99015 (8)	0.19185 (11)	0.27004 (6)	0.0145 (2)	
C18	0.84541 (8)	−0.35806 (11)	−0.16793 (6)	0.0175 (2)	
H18A	0.8628	−0.4291	−0.2020	0.021*	
C12	0.95244 (8)	−0.19665 (11)	0.01989 (6)	0.0151 (2)	
C14	0.72719 (8)	−0.16701 (12)	−0.13212 (6)	0.0188 (2)	
H14A	0.6662	−0.1120	−0.1427	0.023*	
C9	0.85476 (8)	0.25133 (11)	0.18551 (6)	0.0154 (2)	
C2	1.13661 (8)	0.12427 (11)	0.36176 (6)	0.0168 (2)	
H2B	1.1937	0.1780	0.3879	0.020*	
H2C	1.1676	0.0793	0.3176	0.020*	
C13	1.05659 (10)	−0.27421 (13)	0.03706 (7)	0.0226 (2)	
C3	1.02715 (9)	0.34442 (12)	0.38092 (6)	0.0200 (2)	
H3D	1.0163	0.4241	0.3468	0.024*	
H3E	1.0896	0.3646	0.4146	0.024*	
C6	0.69842 (9)	0.30243 (12)	0.09900 (6)	0.0217 (2)	
H6B	0.7367	0.2511	0.0602	0.026*	
H6C	0.6692	0.3872	0.0754	0.026*	
C7	0.75666 (9)	0.47314 (12)	0.20190 (7)	0.0212 (2)	
H7A	0.7753	0.4615	0.2562	0.025*	
H7B	0.6804	0.4975	0.1970	0.025*	
C4	0.92728 (10)	0.32834 (14)	0.42962 (7)	0.0270 (3)	
H4B	0.9165	0.4130	0.4582	0.040*	
H4C	0.9380	0.2512	0.4646	0.040*	
H4D	0.8646	0.3107	0.3967	0.040*	
C5	0.60582 (10)	0.21306 (14)	0.12663 (8)	0.0320 (3)	
H5B	0.5577	0.1901	0.0839	0.048*	
H5C	0.5668	0.2642	0.1643	0.048*	
H5D	0.6344	0.1283	0.1492	0.048*	
C8	0.82384 (11)	0.59180 (13)	0.16992 (8)	0.0321 (3)	
H8A	0.8099	0.6767	0.1976	0.048*	
H8B	0.8045	0.6050	0.1165	0.048*	
H8C	0.8995	0.5687	0.1755	0.048*	
C1	1.09742 (10)	0.01179 (12)	0.41592 (7)	0.0244 (2)	
H1A	1.1571	−0.0478	0.4314	0.037*	
H1B	1.0420	−0.0433	0.3901	0.037*	
H1C	1.0684	0.0553	0.4605	0.037*	
H13A	1.0787 (13)	−0.3377 (19)	−0.0021 (10)	0.043 (5)*	
H13B	1.0561 (14)	−0.325 (2)	0.0845 (11)	0.050 (5)*	
H13C	1.1172 (14)	−0.2076 (19)	0.0445 (10)	0.046 (5)*	
H3A	1.0310 (12)	−0.1104 (16)	0.1438 (8)	0.027 (4)*	

### Atomic displacement parameters (Å^2^)

**Table:**

	*U*^11^	*U*^22^	*U*^33^	*U*^12^	*U*^13^	*U*^23^
N6	0.0158 (4)	0.0174 (4)	0.0152 (4)	0.0012 (3)	−0.0017 (3)	−0.0008 (3)
O2	0.0243 (4)	0.0356 (5)	0.0240 (4)	0.0003 (3)	−0.0093 (3)	0.0004 (4)
O1	0.0308 (4)	0.0284 (4)	0.0169 (4)	−0.0089 (3)	−0.0008 (3)	−0.0064 (3)
N3	0.0177 (4)	0.0185 (4)	0.0148 (4)	0.0038 (3)	−0.0056 (3)	−0.0029 (3)
N5	0.0152 (4)	0.0170 (4)	0.0157 (4)	0.0002 (3)	−0.0001 (3)	−0.0017 (3)
N4	0.0152 (4)	0.0170 (4)	0.0135 (4)	0.0000 (3)	−0.0011 (3)	−0.0009 (3)
N7	0.0160 (4)	0.0182 (4)	0.0143 (4)	0.0002 (3)	−0.0022 (3)	−0.0035 (3)
N2	0.0176 (4)	0.0173 (4)	0.0132 (4)	−0.0008 (3)	−0.0031 (3)	−0.0010 (3)
C19	0.0178 (5)	0.0198 (5)	0.0125 (5)	−0.0063 (4)	−0.0017 (4)	0.0007 (4)
N1	0.0208 (4)	0.0233 (5)	0.0156 (4)	−0.0085 (4)	−0.0025 (3)	0.0010 (4)
N8	0.0173 (4)	0.0187 (4)	0.0196 (4)	0.0037 (3)	−0.0027 (3)	−0.0018 (4)
C15	0.0192 (5)	0.0177 (5)	0.0171 (5)	0.0014 (4)	−0.0011 (4)	−0.0035 (4)
C11	0.0145 (4)	0.0161 (5)	0.0133 (5)	−0.0014 (4)	0.0004 (4)	0.0002 (4)
C16	0.0161 (5)	0.0149 (5)	0.0132 (5)	−0.0017 (4)	0.0000 (4)	0.0010 (4)
C17	0.0166 (5)	0.0161 (5)	0.0179 (5)	0.0003 (4)	0.0008 (4)	−0.0003 (4)
C10	0.0131 (4)	0.0173 (5)	0.0132 (5)	−0.0028 (4)	0.0017 (4)	0.0003 (4)
C18	0.0199 (5)	0.0166 (5)	0.0162 (5)	−0.0033 (4)	0.0024 (4)	−0.0037 (4)
C12	0.0164 (5)	0.0154 (5)	0.0134 (5)	0.0000 (4)	−0.0007 (4)	0.0009 (4)
C14	0.0173 (5)	0.0198 (5)	0.0190 (5)	0.0008 (4)	−0.0025 (4)	0.0005 (4)
C9	0.0141 (5)	0.0170 (5)	0.0150 (5)	−0.0009 (4)	0.0016 (4)	0.0013 (4)
C2	0.0148 (5)	0.0203 (5)	0.0150 (5)	−0.0008 (4)	−0.0027 (4)	−0.0009 (4)
C13	0.0232 (6)	0.0256 (6)	0.0185 (5)	0.0084 (4)	−0.0052 (4)	−0.0043 (4)
C3	0.0209 (5)	0.0204 (5)	0.0184 (5)	−0.0010 (4)	−0.0028 (4)	−0.0064 (4)
C6	0.0205 (5)	0.0249 (6)	0.0192 (5)	0.0062 (4)	−0.0054 (4)	−0.0004 (4)
C7	0.0197 (5)	0.0202 (5)	0.0237 (5)	0.0062 (4)	0.0011 (4)	−0.0026 (4)
C4	0.0279 (6)	0.0320 (6)	0.0213 (6)	0.0027 (5)	0.0039 (5)	−0.0068 (5)
C5	0.0251 (6)	0.0302 (7)	0.0398 (7)	−0.0021 (5)	−0.0131 (5)	0.0053 (5)
C8	0.0366 (7)	0.0212 (6)	0.0388 (7)	0.0008 (5)	0.0071 (6)	−0.0016 (5)
C1	0.0276 (6)	0.0234 (6)	0.0223 (5)	−0.0002 (4)	0.0028 (4)	0.0026 (4)

### Geometric parameters (Å, °)

**Table:**

N6—C11	1.3294 (13)	C12—C13	1.5023 (14)
N6—C9	1.3502 (14)	C14—H14A	0.9300
O2—N1	1.2354 (13)	C2—C1	1.5182 (15)
O1—N1	1.2295 (13)	C2—H2B	0.9700
N3—N2	1.3562 (12)	C2—H2C	0.9700
N3—C11	1.3742 (14)	C13—H13A	0.958 (18)
N3—H3A	0.868 (15)	C13—H13B	0.96 (2)
N5—C10	1.3466 (13)	C13—H13C	0.986 (18)
N5—C9	1.3470 (14)	C3—C4	1.5285 (17)
N4—C11	1.3426 (13)	C3—H3D	0.9700
N4—C10	1.3490 (14)	C3—H3E	0.9700
N7—C10	1.3578 (13)	C6—C5	1.5170 (18)
N7—C2	1.4632 (13)	C6—H6B	0.9700
N7—C3	1.4641 (13)	C6—H6C	0.9700
N2—C12	1.2902 (14)	C7—C8	1.5195 (17)
C19—C18	1.3846 (15)	C7—H7A	0.9700
C19—C14	1.3876 (15)	C7—H7B	0.9700
C19—N1	1.4623 (13)	C4—H4B	0.9600
N8—C9	1.3549 (13)	C4—H4C	0.9600
N8—C7	1.4628 (14)	C4—H4D	0.9600
N8—C6	1.4639 (14)	C5—H5B	0.9600
C15—C14	1.3811 (15)	C5—H5C	0.9600
C15—C16	1.4072 (14)	C5—H5D	0.9600
C15—H15A	0.9300	C8—H8A	0.9600
C16—C17	1.4011 (14)	C8—H8B	0.9600
C16—C12	1.4860 (14)	C8—H8C	0.9600
C17—C18	1.3897 (15)	C1—H1A	0.9600
C17—H17A	0.9300	C1—H1B	0.9600
C18—H18A	0.9300	C1—H1C	0.9600
			
C11—N6—C9	112.92 (9)	C1—C2—H2C	108.9
N2—N3—C11	120.30 (9)	H2B—C2—H2C	107.7
N2—N3—H3A	123.2 (10)	C12—C13—H13A	115.6 (10)
C11—N3—H3A	116.5 (10)	C12—C13—H13B	112.8 (11)
C10—N5—C9	113.82 (9)	H13A—C13—H13B	108.0 (15)
C11—N4—C10	112.86 (9)	C12—C13—H13C	110.4 (10)
C10—N7—C2	120.71 (9)	H13A—C13—H13C	105.4 (14)
C10—N7—C3	120.12 (9)	H13B—C13—H13C	103.7 (15)
C2—N7—C3	119.08 (8)	N7—C3—C4	113.88 (9)
C12—N2—N3	117.98 (9)	N7—C3—H3D	108.8
C18—C19—C14	122.29 (9)	C4—C3—H3D	108.8
C18—C19—N1	119.22 (10)	N7—C3—H3E	108.8
C14—C19—N1	118.47 (10)	C4—C3—H3E	108.8
O1—N1—O2	122.83 (9)	H3D—C3—H3E	107.7
O1—N1—C19	118.75 (9)	N8—C6—C5	111.95 (10)
O2—N1—C19	118.42 (9)	N8—C6—H6B	109.2
C9—N8—C7	121.34 (9)	C5—C6—H6B	109.2
C9—N8—C6	120.77 (9)	N8—C6—H6C	109.2
C7—N8—C6	117.73 (9)	C5—C6—H6C	109.2
C14—C15—C16	121.06 (10)	H6B—C6—H6C	107.9
C14—C15—H15A	119.5	N8—C7—C8	111.88 (9)
C16—C15—H15A	119.5	N8—C7—H7A	109.2
N6—C11—N4	127.98 (10)	C8—C7—H7A	109.2
N6—C11—N3	118.56 (9)	N8—C7—H7B	109.2
N4—C11—N3	113.46 (9)	C8—C7—H7B	109.2
C17—C16—C15	118.28 (9)	H7A—C7—H7B	107.9
C17—C16—C12	122.27 (9)	C3—C4—H4B	109.5
C15—C16—C12	119.44 (9)	C3—C4—H4C	109.5
C18—C17—C16	121.36 (10)	H4B—C4—H4C	109.5
C18—C17—H17A	119.3	C3—C4—H4D	109.5
C16—C17—H17A	119.3	H4B—C4—H4D	109.5
N5—C10—N4	126.06 (9)	H4C—C4—H4D	109.5
N5—C10—N7	116.60 (9)	C6—C5—H5B	109.5
N4—C10—N7	117.34 (9)	C6—C5—H5C	109.5
C19—C18—C17	118.25 (10)	H5B—C5—H5C	109.5
C19—C18—H18A	120.9	C6—C5—H5D	109.5
C17—C18—H18A	120.9	H5B—C5—H5D	109.5
N2—C12—C16	114.46 (9)	H5C—C5—H5D	109.5
N2—C12—C13	123.85 (9)	C7—C8—H8A	109.5
C16—C12—C13	121.66 (9)	C7—C8—H8B	109.5
C15—C14—C19	118.74 (10)	H8A—C8—H8B	109.5
C15—C14—H14A	120.6	C7—C8—H8C	109.5
C19—C14—H14A	120.6	H8A—C8—H8C	109.5
N5—C9—N6	126.22 (9)	H8B—C8—H8C	109.5
N5—C9—N8	117.19 (9)	C2—C1—H1A	109.5
N6—C9—N8	116.59 (9)	C2—C1—H1B	109.5
N7—C2—C1	113.49 (9)	H1A—C1—H1B	109.5
N7—C2—H2B	108.9	C2—C1—H1C	109.5
C1—C2—H2B	108.9	H1A—C1—H1C	109.5
N7—C2—H2C	108.9	H1B—C1—H1C	109.5

### Hydrogen-bond geometry (Å, °)

**Table:**

*D*—H···*A*	*D*—H	H···*A*	*D*···*A*	*D*—H···*A*
N3—H3A···O1^i^	0.868 (15)	2.491 (15)	3.2751 (15)	150.7 (13)
N3—H3A···O2^i^	0.868 (15)	2.474 (15)	3.2486 (15)	149.0 (13)
C2—H2C···N4	0.97	2.38	2.7252 (15)	100
C7—H7A···N5	0.97	2.39	2.7322 (16)	100
C15—H15A···N2	0.93	2.39	2.7128 (15)	100
C1—H1A···Cg2^ii^	0.96	2.91	3.7486 (16)	147
C7—H7B···Cg1^iii^	0.97	2.71	3.3835 (15)	127

Symmetry codes: (i) *x*+1/2, −*y*−1/2, *z*+1/2; (ii) *x*−1/2, −*y*−3/2, *z*−1/2; (iii) −*x*+3/2, *y*+1/2, −*z*+1/2.
